# Supplementary material for: “It’s because I think too much”: Perspectives and experiences of adults with hypertension engaged in HIV care in northern Tanzania
Source: PLoS One. 2020 Dec 3;15(12):e0243059. doi: 10.1371/journal.pone.0243059 (PMC7714125; doi:10.1371/journal.pone.0243059)
Supplement: S2 File — (DOCX) [file pone.0243059.s002.docx]

**S2 File.**

**IN-DEPTH INTERVIEW GUIDE**

**I. INTRODUCTION**

I’ve introduced myself to you, and now I’d like to start by getting to know you a little better. Can you tell me a little about yourself?

[NOTE: Give him/her the opportunity to talk briefly about himself/herself. Let him/her know that you’ll come back to some of these issues later in the interview.]

*Probes*

*Do you have any children?*

*Do you work?*

*Are you married?*

*Where do you live?*

**II. MEDICAL/HIV HISTORY**

Thank you for sharing that with me. I’d now like to ask you a few general questions about your health.

- How old are you?
- Tell me about your health. What medical issues do you have?
- Are you comfortable telling me about your HIV history?

*Probes*

*When were you diagnosed?*

*How were you diagnosed?*

*Where were you diagnosed (i.e. at which health care facility)?*

*Are you on ART?*

*When did you start taking ART?*

*Where were you first prescribed ART (i.e. at which health care facility)?*

*Do you have any problems taking your ART? Why?*

*Do you ever forget to take your ART? Why do you forget?*

*Have you ever stopped your ART? Why?*

*When did you first start going to this CTC clinic?*

*How often do you come to the CTC clinic for appointments or to pick up your medication?*

*Is there anyone that helps you to pick up your medication at the CTC?*

- What other health problems do you have? [*Note: if patient starts talking about hypertension here, note to him/her that you’ll come back to that later for more details.]*
- Have you ever been tested for diabetes?
- Have you ever been tested for kidney disease?
- Have you ever had your eyes checked by a doctor?
- Have you ever had a stroke?
- Have you ever had any problems with your heart?
- Do you smoke cigarettes?
- Do you drink alcohol?
- Other than ART, have you ever been prescribed other types of medication?

*Probes*

*If yes, what type(s) of medication did you use? What was the name? Why did you take this medication?*

- Do you use any herbal or traditional medication?

*Probes*

*What do you use? Why? How long?*

- Where do you receive care for the medical issues that you have other than HIV?

**III. HYPERTENSION DIAGNOSIS**

Let’s start talking more about your high blood pressure now. Tell me about the first time you learned that you had high blood pressure.

- When were you first told you had high blood pressure?
- Who told you that you had high blood pressure?
- What did they tell you?
- Did they tell you what your blood pressure measurements were?
- Where did this happen?
- Why did they check your blood pressure?
- How many times did they check your blood pressure before they told you that you have high blood pressure?
- What was your reaction to the diagnosis?
- Were you diagnosed with high blood pressure first or HIV first?
- What have your healthcare providers told you about your high blood pressure?
- Did you feel that you were helped to understand what high blood pressure is?
- What do *you* think caused your high blood pressure?
- What did your *health* *providers* tell you was the cause of your high blood pressure?
- Do you have any symptoms from your high blood pressure? [IF YES], what symptoms do you have from your high blood pressure?

**IV. FEELINGS AND ATTITUDES ABOUT HYPERTENSION**

Please tell me what it’s been like for your since you first learned that you had high blood pressure.

- How do you feel emotionally about having high blood pressure?
- How has high blood pressure impacted your life?
- Are you concerned about having high blood pressure?
- Does having high blood pressure effect your thoughts about your future?
- Do you feel that high blood pressure is a problem in Tanzania? Why or Why not?
- Do you know of anyone that has high blood pressure?

*Probes*

*If yes, can you tell me what you know about their blood pressure? What do you think of their high blood pressure?*

How does it feel as someone who has both high blood pressure and HIV?

- Do you think your high blood pressure is linked to your HIV? Why or why not?
- Do you think that high blood pressure is linked to ART?
- Do you feel that high blood pressure is a problem among people living with HIV?
- How dangerous is it to have high blood pressure?
- How serious (dangerous) is it to have both high blood pressure and HIV?
- Which is more serious (dangerous), HIV or high blood pressure? Why?

**V. KNOWLEDGE OF HYPERTENSION**

I’d now like to talk to you about what you know about high blood pressure. To begin, can you tell me what you know or have been told about high blood pressure.

- What does it mean if someone has high blood pressure? *(What do you mean by the term pressure? Or What is high blood pressure?)*
- What is a normal blood pressure measurement?
- What is a high blood pressure measurement?
- What are some of the causes of high blood pressure?

*Probes*

*What is the most common cause?*

*Do you think stress is related to blood pressure? How?*

*Is alcohol linked to high blood pressure?*

*Is smoking linked to high blood pressure?*

*Can high blood pressure be passed down through the family?*

*Is weight linked to high blood pressure?*

*Is age linked to blood pressure?*

*How do you think these things cause (produce) high blood pressure?*

- What are some of the symptoms a person with high blood pressure may have? (*What are some things that may indicate that a person has high blood pressure?)*

*Probes*

*What is the most common symptom?*

*Do you think it is possible for someone with high blood pressure to not have any symptoms?*

- What are some of the risks (complications) of having high blood pressure? *(What effects do high blood pressure have on the body?)*

*Probes*

*What is the most serious risk (complication) of having high blood pressure?*

- What are ways to control high blood pressure?

*Probes*

*What do you think is the most effective way to control high blood pressure?*

*What are some ways to control high blood pressure without using medication prescribed by a doctor?*

*Stress reduction techniques? What are ways to reduce stress?*

*Have you heard of losing weight to control blood pressure?*

*Have you heard of making dietary changes to control blood pressure? What dietary changes?*

*Have you heard of using medication to control high blood pressure?*

*Have you heard of using herbal treatment or traditional medication to control blood pressure?*

- What are some concerns people may have about using blood pressure medication?
- Can high blood pressure be cured? How?
- Can you prevent high blood pressure? How?

**VI. HYPERTENSION CARE**

Let’s talk about the care you’ve received for your high blood pressure.

- Where do you get your care for your high blood pressure?

*Probes*

*Have you ever received care for your high blood pressure anywhere else?*

- How often do you see a health care provider for your high blood pressure?
- Do you expect you will always have high blood pressure?

*Probes*

*Who told you? What did they tell you? How do you feel about this?*

- Have you received any education about your high blood pressure?

*Probes*

*Who provided the education?*

*What was discussed?*

*How was the attitude of the provider?*

*Were all your questions answered?*

*Did you understand everything that was discussed about your high blood pressure?*

*How often do you receive education sessions about high blood pressure?*

- Do you feel knowledgeable about high blood pressure?
- How do you feel about the care you have received for your high blood pressure?

**VII. HYPERTENSION TREATMENT**

Now, let’s talk about some of the ways that your blood pressure has been controlled. What are some of the ways that your high blood pressure has been reduced?

- What kinds of things have you personally done to improve your high blood pressure?
- Tell me what usually happens when you are seen in the clinic for your high blood pressure?

*Probes*

*Has a health provider ever discussed ways to control your high blood pressure with you?*

*[IF YES], what did they discuss with you? medications, lifestyle modification (i.e. exercise, weight loss, diet changes, salt reduction, alcohol cessation, smoking cessation, stress reduction)*

*Which of these things did they recommend for you?*

*Did you follow their recommendations? [If yes], what did you do? [If no], why not?*

*If you followed their recommendations, do you think that it helped to improve your blood pressure? Why or why not?*

*How often does your health provider discuss ways to control blood pressure with you?*

*How long were you told you need to be taking care of your high blood pressure?*

*Have your health providers helped you to understand the risks and benefits of taking care of your high blood pressure*

- Has your health provider referred you to any specialists (i.e. ophthalmologist, nephrologist, endocrinologist, cardiologist)? [If yes], why?
- Have you ever experienced any medical complications from your high blood pressure?

*Probes*

*Since you have known you have high blood pressure, Has your health provider ever asked you to do investigations at the lab (i.e. blood work)?*

*[If yes], do you know what type of blood work?*

*How often do you get blood work done?*

*Do you know why your health provider wants you to have blood work done?*

*Do you know what the results were?*

*Has your health provider ever discussed the results with you?*

- Have you ever used medications for your blood pressure? **IF NO, CONTINUE TO SECTION A. IF YES, SKIP TO SECTION B.**

**A. IF THE PATIENT HAS *NOT* TAKEN MEDICATIONS FOR BLOOD PRESSURE:**

- Why haven’t you used medications for your blood pressure?
- How do you feel about using medications for blood pressure?
- What are your feelings about using a medication for the rest of your life?
- Have you ever *not* used medication for high blood pressure that was recommended to you by your health provider? [IF YES], what were your reasons for not using the medication?

**SKIP TO SECTION C**

**B. IF THE PATIENT HAS TAKEN MEDICATIONS FOR BLOOD PRESSURE:**

- What was the name of the medication(s)?
- Who prescribed the medication for you?
- Where did you receive the prescription?
- How many times a day did you use the medication?
- Do you have insurance for getting medication in the clinic?
- [If no], Where did you purchase the medication?
- How much did the medication cost?
- How long did you take the medication for?
- Did you ever miss a dose of the medication? [If yes], why did you miss the dose?
- Have you ever stopped taking your blood pressure medication? [If yes], why did you stop? When did you stop? For how long did you stop?
- Are you still taking the medication or have you stopped taking it?
- What have you been told about how long you need to be on medications for high blood pressure?
- Have you experienced any complications from using the blood pressure medication?
- Have you ever not taken medication for high blood pressure that was recommended to you by your health provider? [IF YES], what were your reasons for not taking the medication?
- What are your feelings about taking medication for high blood pressure?
- What are some concerns you have about taking medication for blood pressure?
- What are your feelings about taking a medication for the rest of your life?
- What are some concerns you have about taking medications for both your high blood pressure and your HIV?

**C. TRADITIONAL AND ALTERNATIVE TREATMENT:**

- Other than medication is there anything else that you have done to try and reduce your blood pressure?

*Probes*

*Where did you hear about this way to reduce your blood pressure?*

*Do you think it helped you?*

- Have you ever taken traditional medication for your blood pressure?

*Probes*

*What did you take?*

*How long did you take it?*

*Did it help you?*

*Why did you decide to take traditional medication?*

*Have you ever seen a traditional healer for your blood pressure?*

*Did you take the traditional medication and the blood pressure medication you received from the hospital at the same time?*

**VIII. HIV AND HYPERTENSION CARE**

Now I’d like to spend some time talking about both your HIV and high blood pressure.

- What’s it like to be a person living with HIV and with high blood pressure?
- Does having *HIV* affect the health care you’ve received for your high blood pressure? If yes, in what way?
- Does having *high* *blood* *pressure* affect your HIV care? If yes, in what way?
- Do you think that having HIV has made it more difficult for you to receive health care for your high blood pressure? If yes, how so and why?
- Does the same health care provider/clinic manage both your HIV and high blood pressure or is it separate?

*Probes if separate:*

*Does your high blood pressure provider know that you have HIV?*

*Does this provider ever discuss your HIV with you?*

*Does this provider ever ask you what other medications you are taking?*

*Does this provider ever ask if you have other health issues besides high blood pressure?*

- Does your *HIV* provider know that you have high blood pressure?

*Probes*

*Has your HIV provider ever discussed your high blood pressure with you?*

*If so, how often? What does she/he discuss with you?*

*Does your HIV provider ever ask if you are taking other medications besides ART?*

*Does your HIV provider ever ask you if you have other health issues besides HIV?*

- Is your blood pressure ever checked in the HIV clinic?

*Probes*

*If yes, how often is it checked in the HIV clinic?* Would you like to receive both your HIV and high blood pressure care at the same CTC clinic visit? *Why* *or* *why* *not?*

*Would you feel comfortable if your HIV provider also managed your blood pressure? Why or Why not?*

- Do you talk to other people about your high blood pressure?

*Probes*

*[If yes],how did they react? What did the people you told say to you?*

*[If yes], who have you told?*

*What have you told them?*

- What are people’s reactions if they find out that you have high blood pressure?
- Do you talk to other people about your HIV?

*Probes*

*If yes, who have you told?*

*What have you told them?*

*What are people’s reactions when they find out you have HIV?*

**IX. BARRIERS TO IMPROVED CARE**

Sometimes there may be challenges that arise that make it difficult to receive the medical care you need.

- What are some challenges that you’ve faced that have prevented you from receiving care for your blood pressure?
- What are some things that prevent you from going to the clinic? Are you always able to make it to clinic on your appointment date? If not, why?

*Probes*

*Transportation issues*

*Cost of transportation*

*Can’t make appointment for other reason (childcare, employment, etc.)*

*Stigma of being seen at clinic*

- What are things that prevent you from receiving care for your blood pressure when you are at the clinic? (*When you’re at the clinic what challenges are you facing?)*

*Probes*

*Blood pressure is not checked at clinic*

*Medications are not available at the clinic*

*Long wait times at the clinic*

*Cost of medications, cost of clinic visits, cost of labs*

- What are some challenges you have with healthcare providers?

*Probes*

*Healthcare provider doesn’t discuss or prescribe treatment for blood pressure*

*Not enough healthcare providers*

*Healthcare provider doesn’t spend a lot of time with patients*

*Healthcare provider has poor attitude*

- What are some challenges you face with getting medication or using medication?

*Probes*

*Don’t want to take medication*

*Medication too expensive*

*Side effects from medication*

- What are some financial challenges you have when it comes to taking care of your blood pressure and your health?

*Probes*

*Costs of transportation, clinic visit, labs or medications are too high*

- What are some challenges of having HIV that effect your blood pressure care?

*Probes*

*Stigma related to HIV*

*Interaction between ART and blood pressure medications*

- What do you think the general public feel about having high blood pressure? What do you think the general public feel about treatment for high blood pressure?

*Probes*

*Belief in cure*

*Mistrust/doubt of diagnosis*

*Attitudes about treatment*

*Stigma associated with high blood pressure*

- What would you want to change about the care you have received for your high blood pressure?
- What would you want to change about the care you receive for your HIV?

**X. DESIRED SEVICES AND FACILITATORS TO IMPROVED CARE**

- What other information would you like to get about your high blood pressure?
- What types of questions do you have about high blood pressure?
- What are some things that health providers could do to help you with your blood pressure?
- What are some other services that would you like to receive for your high blood pressure in the future?
- What does having well controlled blood pressure mean to you?

**XI. CONCLUSION**

Our interview is coming to an end.

- Do you have anything else you’d like to add or share before the interview is over?

Thank you very much for taking the time to talk with me. Your views and experiences will help us to care for people with high blood pressure and HIV in Tanzania.
