# Supplementary material for: “It’s because I think too much”: Perspectives and experiences of adults with hypertension engaged in HIV care in northern Tanzania
Source: PLoS One. 2020 Dec 3;15(12):e0243059. doi: 10.1371/journal.pone.0243059 (PMC7714125; doi:10.1371/journal.pone.0243059)
Supplement: Attachment — Submitted filename: Manuscript_Hypertension & HIV wm.docx [file pone.0243059.s004.docx]

“It’s because I think too much”: Perspectives and experiences of adults with hypertension engaged in HIV care in northern Tanzania

Preeti Manavalan^1,2^*, Linda Minja^3^, Lisa Wanda^3^, Julian T. Hertz^2,4^, Nathan M. Thielman^1,2^, Nwora Lance Okeke^1^, Blandina T. Mmbaga^2,3,5^, Melissa H. Watt^2,6^

^1^Division of Infectious Diseases, Department of Medicine, Duke University Medical Center, Durham, NC USA

^2^Duke Global Health Institute, Durham, NC USA

^3^Kilimanjaro Clinical Research Institute, Moshi, Tanzania

^4^Division of Emergency Medicine, Department of Surgery, Duke University Medical Center, Durham, NC USA

^5^Kilimanjaro Christian Medical University College, Moshi, Tanzania

^6^Department of Population Health Sciences, University of Utah, Salt Lake City, UT USA

***Corresponding Author:**

Preeti Manavalan MD

E-mail: preeti.manavalan@gmail.com

**Header:** HIV and hypertension in northern Tanzania

**Abstract**

**Background:** Hypertension, a leading risk for cardiovascular mortality, is an important co-morbidity among people living with HIV (PLHIV). In Tanzania, hypertension prevalence among PLHIV approaches 20 to 30%. Despite this high burden of disease, most patients are unaware of their diagnosis and are not receiving treatment. Understanding the barriers to hypertension care is a critical first step in developing interventions to improve cardiovascular outcomes among PLHIV in Tanzania and similar settings.

**Methods:** Between September 1^st^ and November 26^th^, 2018 thirteen semi structured in-depth interviews were conducted with hypertensive patients engaged in HIV care in two HIV clinics located in government health facilities in northern Tanzania. Participants were purposively selected until thematic saturation was reached. Data analysis was conducted using applied thematic analysis.

**Results:** Participants ranged in age from 28 to 78 years, with a median age of 54 (IQR 41 – 65) years. Of the 13 participants, eight stated they had used antihypertensive medication previously, but only one participant described current use of antihypertensive therapy. All participants were currently using antiretroviral therapy. The data revealed a range of themes including limited poor hypertension knowledge. Universally, all participants believed that “thinking too much”, i.e. stress and having worries, was the major contributor to hypertension and that by “reducing thoughts”, one may control hypertension. Additional emerging themes included a perceived overlap between hypertension and HIV, delays in hypertension diagnosis and linkage to care, challenges with provider communication and counseling, reluctance to use antihypertensive medication, lack of integration of hypertension and HIV care, and other barriers to hypertension care.

**Conclusions:** Participants described multiple, intersecting challenges related to hypertension management. Multifaceted strategies that address these barriers and that are integrated within HIV care are urgently needed to improve cardiovascular outcomes among PLHIV in sub-Saharan Africa.

# Introduction

With scale-up of antiretroviral therapy (ART), HIV-related mortality has declined rapidly in the last decade, including in sub-Saharan Africa (SSA) [1]. People living with HIV (PLHIV) are now living longer and are faced with a double burden of disease: HIV infection and chronic non-communicable diseases (NCDs), including cardiovascular disease (CVD) [2,3]. CVD is the most common cause of death globally [4], and in PLHIV it occurs earlier and more frequently. In comparison to adults without HIV infection, PLHIV have a 50% increased risk for myocardial infarction and stroke and a 4-fold higher risk of sudden cardiac death [5,6]. Hypertension is a major risk factor for CVD and is the leading risk for death world-wide [7], and it is estimated that up to a third of adults with treated HIV meet criteria for hypertension [8].

SSA has the highest prevalence of hypertension in the world [9], and in Tanzania approximately 1 in 3 people may be hypertensive [10]. Despite this increased burden of disease, most patients with hypertension in SSA are not on treatment. A meta-analysis examining hypertension in SSA revealed that 73% of hypertensive adults were unaware of their diagnosis, 82% with a known diagnosis were not on treatment, and 93% of those on treatment had an uncontrolled blood pressure [11]. These concerning trends are also present among persons with HIV [12]: a recent study in northern Tanzania found that among a cohort of hypertensive patients enrolled in HIV care, 65% were unaware of their diagnosis, 90% were not on antihypertensive treatment and all had uncontrolled blood pressure [13].

Several observational and qualitative studies have identified barriers to high quality hypertension care among the general patient population in SSA. Impediments to care include lack of access to blood pressure equipment and drugs, absence of guidelines for hypertension management, shortages in clinical staff, gaps in provider and patient knowledge, financial constraints, and patient mistrust in the health care system. [14–16]. Whether PLHIV face unique barriers to hypertension care remains unclear. However, the challenges faced by hypertensive adults with HIV may be different compared to the general population. PLHIV have more non-traditional risk factors for CVD compared to their HIV-negative counterparts. Even without major CVD risk factors, HIV-infected adults have a twofold increased risk of myocardial infarction [3]. In addition, PLHIV may be unlikely to seek care outside of the HIV clinical setting and must often navigate perceptions of disease-related stigma [17,18].

In order to design interventions that address challenges unique to this patient population, it is crucial to understand the experiences of PLHIV who have navigated hypertension treatment. However, other than a few studies [19–23], the barriers to hypertension awareness, treatment and control among HIV-infected adults in SSA remain largely unexplored. To better understand barriers to hypertension management among PLHIV, we conducted a qualitative study exploring the perspectives and experiences of patients with both HIV and hypertension in northern Tanzania.

**Methods**

This qualitative study involved in-depth interviews with 13 individuals who were actively involved in HIV care and reported a history of hypertension. To ensure rigor and reproducibility, the presentation of methods and results follow the Consolidated Criteria for Reporting Qualitative Research (COREQ) guidelines (S1 File) [24].

**Setting**

This study was situated in the Moshi urban district of northern Tanzania. In northern Tanzania, the approximate community prevalence of hypertension among adults is 28% [25]. The study was conducted at two HIV clinics located in government-funded primary health centers, Majengo Health Center and Pasua Health Center. Majengo Health Center sees approximately 1200 adults (900 women and 300 men) with HIV per year and Pasua Health Center sees approximately 1100 adults (800 women and 300 men) with HIV per year. In both health centers, HIV care is typically provided by nurses and clinical officers in the HIV Care and Treatment Center. Hypertension care is managed separately from HIV care, and is managed by a medical doctor or clinical officer in a different department.

**Study population**

Semi-structured in-depth interviews were conducted with 13 patients with HIV and with a history of hypertension. Participants were eligible if they were active patients in one of the two HIV clinic study sites and had a self-reported diagnosis of hypertension as reflected in the criteria outlined below. Data collection was carried out until thematic saturation was reached.

**Procedures**

Eligible participants with known HIV and hypertension were recruited by the HIV clinic and research staff between September 1^st^ and November 26^th^, 2018. During their routine HIV appointments patients were asked by the HIV clinic nurse or by the study nurse if they: 1) had a known diagnosis of hypertension, 2) were ever told by a health provider they had high blood pressure, and 3) had ever used medications to control blood pressure. If a patient met any of these criteria and also reported using ART for a minimum of 12 months, then the study nurse invited the patient to participate in the study.

The in-depth interview guide was developed by an interdisciplinary team of physicians, nurses and social scientists from Tanzania and the United States with expertise in hypertension or HIV. The guide included open ended questions on key domains of interest (medical history, hypertension diagnosis, feelings and attitudes about hypertension, knowledge of hypertension, hypertension care and treatment, HIV and hypertension care, barriers to improved care, and desired services and facilitators to improved care), with each question followed by a list of possible follow-up probes to guide the conversation (S2 File). The guide was reviewed by Tanzanian collaborators to ensure appropriate language, terminology and cultural saliency. All interviews were conducted in a private room at the study site by a female physician researcher from the US with graduate level training in qualitative methodology (PM), who was not involved in any clinical care at the study clinic sites. The interviews were conducted in English and were simultaneously translated in Swahili in real-time by a female Tanzanian research assistant (LM) who had received training in qualitative methodology. Interviews lasted 59 to 157 minutes with a median duration of 105 minutes, were audio recorded with participant consent, and were subsequently transcribed in English. A debriefing meeting was held between the interviewer (PM) and the research assistant translating the interviews (LM) following every interview. During the debriefing meetings preliminary themes were identified and new information added from each interview was discussed. A third member of the research team (JTH) listened to 15% of all audio recordings to confirm and clarify the on-site translation.

**Data analysis**

Data analysis was conducted using applied thematic analysis [26] guided by a phenomenology theoretical framework, which is well suited to understanding individuals’ lived experiences and the subjective nature of their health seeking processes [27,28]. As a first step, transcripts were reviewed, and memos (5-19 pages each) were written for each transcript to summarize the content emerging across the domains of interest [29]. After reviewing all memos, a codebook was developed that consisted of 10 parent codes and 11 child codes. The transcripts were independently coded in NVivo by a physician researcher from the US (PM) to label the transcript content with the respective themes. A Tanzanian member of the research team (LW) double-coded 3 (20%) of the transcripts, and the two team members met to discuss coding protocol, reach consensus on the application of codes, and make appropriate modifications to the code book. Coding queries were used to review text coded to themes and synthesize data. Representative quotes were reviewed throughout the analysis procedure and were selected to best capture the data.

**Ethics**

Study procedures were approved by Duke Health Institutional Review Board (#XXX), Kilimanjaro Christian Medical Centre Research Committee (#XXX), and the Tanzania National Institute for Medical Research Ethics Coordinating Committee (#XXX). All participants were introduced to the physician researcher from the US (PM) and the Tanzanian research assistant who served as translator (LM), and were made aware of the overall purpose of the study during consent procedures. All participants provided written informed consent prior to data collection. All participants were compensated approximately $2.20 USD (5000 Tanzanian shillings) as reimbursement for their time.

**Results**

The characteristics of study participants are summarized in Table 1. Participants had a median age of 54 (IQR 41 – 65) years. The sample included 11 women and 2 men. Most participants had some primary school education. The median duration of time since HIV diagnosis and duration of time of ART use was 4 (IQR 3.0 – 10.0) years and 3 (IQR 2.5 – 6.5) years, respectively. Ten participants had been diagnosed with hypertension after their diagnosis of HIV. No participant was diagnosed with hypertension in the HIV clinic; eight participants were diagnosed in the outpatient department (OPD), an urgent care clinic, two were diagnosed at a community health fair, one during a hospitalization, one in the preoperative setting, and one at a local pharmacy. In addition to hypertension and HIV, ten participants also reported having another chronic medical condition (including diabetes, dyslipidemia, stroke, and chronic ocular, genitourinary, gastrointestinal and cardiopulmonary issues). Of the 13 participants, eight stated they had used antihypertensive medications previously. At the time of the interview, only one participant described current use of antihypertensive therapy.

**Table 1. Characteristics of in-depth interview participants, Kilimanjaro Region, 2018 (n=13)**

| **Characteristic** | **n** |
| --- | --- |
| Sex |  |
| Female | 11 |
| Male | 2 |
| Age |  |
| 18 – 35 | 1 |
| 36 – 50 | 5 |
| 51 – 70 | 5 |
| 71+ | 2 |
| Marital status |  |
| Single | 1 |
| Married | 2 |
| Separated/divorced | 3 |
| Widowed | 7 |
| Education |  |
| None | 3 |
| Primary (grade 0 – 8) | 9 |
| Secondary or higher (grade 9 or higher) | 1 |
| Median duration of time since HIV diagnosis, years (IQR) | 4.0 (3.0 –10.0) |
| Median duration of ART use, years (IQR) | 3.0 (2.5 – 6.5) |
| Median duration of time since hypertension diagnosis, years (IQR) | 1.0 (0.0 – 3.0) |
| Ever used antihypertensives |  |
| Yes | 8 |
| No | 5 |
| Currently using antihypertensives |  |
| Yes | 1 |
| No | 12 |
| Other chronic illnesses |  |
| Yes | 10 |
| No | 3 |

ART: antiretroviral therapy

The data revealed a range of themes across the domains of perspectives and experiences of hypertension care (see Table 2).

**Table 2. Dominant themes of hypertension care among patients with HIV and hypertension, Kilimanjaro region, 2018 (n=13)**

| **Domain** | **Themes** |
| --- | --- |
| **Perspectives** | **Limited knowledge of hypertension**  **Poor understanding of causes, mitigation, progression, management**  **Limited understanding of the causes of hypertension** |
|  | Belief that “thinking too much”, i.e. stress, is the major cause |
|  | Belief that HIV treatment and worrying about one’s HIV status is a cause |
|  | Lack of knowledge of some lifestyle factors such as sedentary lifestyle, obesity, smoking, and alcohol use as a cause |
|  |  |
|  | Poor understanding of definition, course of illness, symptoms, complications, and treatment |
|  | Source of knowledge from lay-beliefs from the community |
|  | **Perceived overlap and comparisons between hypertension and HIV** |
|  | Feeling overwhelmed with having more than one medical condition |
|  | Belief that HIV is chronic and controllable versus hypertension is temporary and fatal |
|  | Belief that HIV requires a daily life-long medication versus hypertension is curable |
| **Experiences** | **Delays in hypertension diagnosis and linkage to care** |
|  | Diagnosis made only after feeling ill and presenting with symptoms |
|  | Delay in linkage to care following diagnosis |
|  | **Challenges with provider communication and counseling** |
|  | Limited counseling from providers |
|  | Misconceptions often communicated to patients |
|  | **Reluctance towards antihypertensive medication** |
|  | Preference for stress reduction, traditional treatment and lifestyle modification |
|  | **Lack of integration for hypertension and HIV care** |
|  | Separation of care |
|  | HIV clinic as entry point into hypertension care |
|  | **Additional barriers to hypertension care** |
|  | High cost of care |
|  | Drug side effects |
|  | Lack of access to antihypertensive medication |
|  | Staff shortages and long wait times |
|  | HIV associated stigma and social isolation |

**Perspectives of hypertension**

**Limited knowledge of hypertension**

Overall discussions revealed poor knowledge of hypertension, including what it is, its causes, mitigation, progression and management. All participants perceived their cause for hypertension to be due to ‘thinking too much’ and many believed this was the sole cause of their hypertension. Thinking too much included ‘being angry’, ‘annoyed or upset about something’, ‘receiving shocking news’, ‘having a lot of worries’, or ‘being stressed’. As explained by two interviewees:

“When you hear about shocking news, like the death of someone who is very close to you, that can lead to high blood pressure. Or if your family has problems, if they are annoying you, or disturbing you, or giving you problems that may cause high blood pressure.” (Female, 45 years)

“Primarily stress (causes hypertension). If you think a lot, you will get that problem.” (Female, 28 years)

Overall, participants believed worrying about their HIV status or being stigmatized from HIV could lead to hypertension. A 54-year old woman explained:

“If you accept your HIV results and you are at peace with them, then you won’t have high blood pressure. But if you don’t accept your results, then your blood pressure can be high.” (Female, 54 years)

A few respondents vocalized concern that their ART may be the cause of their hypertension due to adverse side effects. As explained by a 40-year old woman:

“I once asked the (HIV) doctor about the medication (ART). Like see, ‘I have pressure. Do you think the medication (ART) I am using is what’s causing it?’…I was worried, because before I started using the HIV medication I didn’t have high blood pressure.” (Female, 40 years)

All but 1 participant identified an unhealthy diet high in salt, fat and oil as a cause for hypertension. Few participants identified sedentary lifestyle and obesity as a cause. Only 1 participant identified alcohol use as a hypertension risk factor and no participant reported tobacco use as a cause. Chemicals and pesticides used in farming were also believed to be potential causes of hypertension.

Participants demonstrated limited understanding of hypertension. No respondent was able to correctly define hypertension. When asked, most participants defined high blood pressure as what they perceived as the cause: ‘thinking a lot’, ‘being stressed’ or ‘receiving shocking news’. A few participants also described high blood pressure as what they perceived as the symptoms: ‘heart beating too fast’, the heart becoming ‘tired’ or ‘weak’, or ‘blood flow’ abnormalities.

Participants were unaware of the range of hypertensive values and the chronicity of hypertension; instead, they believed that hypertension was more of a ‘temporary problem’. While participants often self-identified as being asymptomatic, most assumed it was unusual to have hypertension and be asymptomatic. Commonly described hypertension symptoms included headache, dizziness, shortness of breath and palpitations. Patients felt that being aware of their symptoms was sufficient enough to tell if their blood pressure was high. Perceived complications from hypertension included instantaneous death, falling down suddenly, rupture of blood vessels leading to instantaneous death, and stroke. As explained by several participants below:

“You might just fall anywhere (from hypertension). You may fall in the middle of the road while you are by yourself.” (Female, 63 years)

“With pressure, if it goes up suddenly you will die, because I have seen that. That’s what happened to my mother. Suddenly her blood pressure became so high and she didn’t receive any help. The doctors later told us after the postmortem that due to high blood pressure some of the blood vessels near her neck burst and she died.” (Female, 45 years)

“It (hypertension) will kill you instantly. …I can give you an example of what happened to my neighbor. One day he just fell and when they went to help him they found that he had a stroke. And I asked, ‘What is stroke?’. They said, ‘Stroke is pressure’. And I said, ‘Oh no’. So high blood pressure can also do this to you.” (Female, 55 years)

All participants believed that hypertension could be controlled by reducing one’s worries. In addition, five participants believed that using herbal or traditional treatment could control blood pressure, three participants believed that temporary use of antihypertensive medication could cure hypertension, and two participants believed that hypertension could be cured through prayer. Reducing stress was believed to be the most effective way to treat hypertension. One participant who trained as a nursing assistant reported that she was taught in nursing school that stress is the most common cause of hypertension and believed that stress reduction could cure hypertension. Another woman explained:

“To reduce blood pressure, maybe someone should avoid (environmental) noise and try to reduce stress in their lives and conflicts…When there is a problem you know you can’t solve, you should just try not to think about it. Like me for example, I want to build a house, but I can’t buy bricks. I can’t buy the cement. I can’t buy the sand I need. So, I don’t want to constantly think about it. I just ask God, please help me with this because in the meantime I can’t handle this.” (Female, 45 years)

Participants appeared hesitant about using medication to control blood pressure and expressed a preference for alternatives to pharmacological treatment. Participants were unaware that antihypertensive medication use was generally life-long. All but one respondent was able to identify some element of lifestyle modification that could improve blood pressure including reduction of salt, fat and oil. Participants were largely unaware that weight loss and alcohol and smoking cessation could reduce blood pressure.

All participants reported learning about hypertension from other members of the community including hypertensive family members, friends, neighbors or co-workers. Information about hypertension was typically acquired through hearsay or from personal experiences. Only a few participants cited learning about hypertension from a medical provider. Most participants vocalized that they would like more information about hypertension.

**Perceived overlap and comparisons between hypertension and HIV**

Patients expressed concern about having both HIV and hypertension and described having two illnesses as ‘overwhelming’. They alluded to pill burden and felt that taking two medications for two diseases was ‘tiresome’ and ‘too much’. One interviewee stated:

“It’s tiresome. It’s really tiresome because I have to take antiretrovirals every day, every single day. And adding another medication is tiresome.” (Female, 55 years)

A few participants reported that they would be willing and even ‘happy’ to use antihypertensive medications every day because they were used to taking ART. While HIV was universally perceived as a chronic disease that was controllable, most participants believed that hypertension was temporary but almost always fatal. Hypertension was believed to be more dangerous than HIV because it could lead to instantaneous death, whereas HIV could be controlled with medication and one could still live a long and healthy life. A 45-year old woman stated:

“I think it’s better to have HIV than pressure…You know with HIV you can just take medication and you will be fine. You can just continue with your life. But with pressure it can just go up suddenly and you can die instantly…I saw that with my aunt. It started in the night and in the morning they were dressing her to take her to the hospital and she died. That’s why I’m scared.” (Female, 44 years)

Participants believed that there were alternative options to antihypertensive drugs for blood pressure control, whereas they believed the only way to control HIV was through ART. A 55-year old woman stated:

“I’ve never thought about stopping using my HIV medications because I know I have to use it for the rest of my life... They explained it clearly at the clinic, ‘There’s no day that you’re going to be cured (from HIV). So, we’re giving you this medication for you to live as long as possible. You should not stop it, because once you do, you will be at risk for opportunistic diseases like tuberculosis and other things like that’. So I’ve never thought about stopping the HIV medication. But for high blood pressure? I guess maybe there will be a cure or an alternative.” (Female, 55 years)

**Experiences of hypertension care**

**Delays in hypertension diagnosis and linkage to care**

Blood pressure was usually only measured when participants felt ill and presented to a health facility with symptoms, such as a headache, dizziness, fatigue or insomnia. All participants reported being diagnosed with hypertension when they presented with perceived symptoms. One interviewee described how she only checks her blood pressure when she feels unwell:

“I check my blood pressure once in a while because sometimes I may receive some scary news and hear things that worry me. And when things like that happen my heart starts beating fast and I get short of breath. When I feel like that I go to the dispensary and check my pressure.” (Female, 45 years)

Several participants noted a delay in linkage to care following their initial hypertension diagnosis or were not linked to care at all. One patient, a 67-year old man, stated he first heard his blood pressure was high more than 3 years ago when his blood pressure was measured in the pre-operative setting. At the time, he was told his surgery was canceled due to his high blood pressure, but he received no counseling, linkage to care or management for his hypertension. He reported only becoming aware that he had hypertension within the past year when he presented to his HIV clinic with a headache and was referred to the OPD for a blood pressure check.

“They wanted to do the operation on the eye and I went to the hospital. When I was waiting for the operation, they checked my blood pressure and they said, ‘No, this blood pressure is so high. We can’t operate when your blood pressure is so high’…They didn’t tell me anything (about my high blood pressure). They just said that they couldn’t do the operation…I was never told again (about high blood pressure).” (Male, 67 years)

**Challenges with provider communication and counseling**

Respondents reported receiving minimal counseling from their healthcare providers regarding the causes, symptoms, complications and treatment of hypertension. When medications were prescribed, respondents noted they did not receive counseling regarding side effects, potential drug-drug interactions, treatment duration or medication adherence. Most respondents cited receiving lifestyle modification counseling including salt reduction, eating a healthy diet, exercise and stress reduction. Participants reported that healthcare providers often did not discuss blood pressure monitoring and follow-up plans for hypertension.

In addition, participants described hypertension misconceptions communicated by their medical providers. Participants conveyed that healthcare providers told them that ‘thinking too much’ was the cause of their hypertension. Some respondents said that their providers told them to stop worrying about their HIV, and often asked them what they were thinking about when their blood pressure was high. One woman who had evidence of pre-eclampsia and suffered a subsequent miscarriage described being told by her provider that her hypertension was due to her grief. Another woman described how the nurse implied that her hypertension was due to conflicts with her husband.

“He (the doctor) didn’t discuss much about the cause but he told me that it may be because of thinking too much and being stressed out. The nurse even asked me, ‘Do you have problems with your husband?’ And I said, ‘Yes Nurse, I’ve had an argument again’, and she said, ‘Okay you should not think too much about these things’.” (Female, 39 years)

Some participants referred to situations where a provider advised them either not to initiate antihypertensive medications or advised them to stop and ‘take a rest’ from their medication because their blood pressure was now in a normal range or because they were asymptomatic.

“I was feeling fine. So when I went back (to the clinic) the doctor was very happy, and he said, ‘Wow, you are doing great. I am happy that you are doing great. So let’s see if you can stay without the (antihypertensive) medication for 2 weeks. I want you to be without the medication for 2 weeks and then we will see.’” (Female, 40 years)

“For this hypertension medication, they told us that if you’re feeling better you can stop the medication. The nurses told me.” (Male, 67 years)

**Reluctance towards antihypertensive medication**

While over half of participants reported receiving a prescription for antihypertensive drugs at some point in their lives, most participants were reluctant to use pharmacologic therapy and preferred non-pharmacologic approaches to blood pressure control. Participants who reported having received a prescription typically only used their antihypertensives when feeling symptomatic or until their prescription ran out. Reasons for discontinuing medication included high costs, lack of availability at the local pharmacy, drug side effects, pill burden from taking too many medications, and lack of awareness about the silent and chronic nature of disease. One participant explained:

“But you know as a human sometimes when I feel like I’m okay I don’t take the (antihypertensive) medication. But when I get sick that’s when I say, ‘Okay, now I know I have to take the medication’…So, since 2002 up until now, I know the doctor told me I should always take (antihypertensive) medication, but I can’t afford that. So I usually only take the medication when I feel I’m sick…When I don’t have money to buy the medication and I’m not feeling good I drink a lot of water because they told me it will help and I eat a lot of cucumbers.” (Female, 63 years)

At the time of this study, only one participant was still using antihypertensive drugs. All other respondents were either not using any methods for blood pressure control or were using stress reduction, herbal or traditional treatment or lifestyle modification with the intention to ‘cure’ their hypertension. All respondents reported that they tried to ‘reduce their thinking’, i.e. decrease stress and worrying, in order to control their blood pressure. A few participants had tried herbal, natural and traditional treatment including drinking lots of water, eating cucumbers, using herbal plants, using ginger and garlic supplements, and also visiting a traditional healer. Participants who used traditional and herbal treatment typically stopped using their antihypertensive drugs and did not discuss the use of their herbal treatment with their clinical providers. Two participants described their preference for herbal and natural medications below:

“I’m using some herbal medication. This is the person I have seen (a traditional healer). He was able to cure people with cancer and with goiters…he told me that I have to use the (traditional) medication for a while. The medication is only one teaspoon three times a day…I’m not sure what is in there, but it seems like it is several things that are combined because it is in the form of a powder. I can’t use these medications together with the hospital medication so I stopped the hospital medication (antihypertensives).” (Female, 40 years)

“I thought it would be too much for me. I already have this medication (for HIV), and now starting this medication (for hypertension) that I have to take for a long time? I didn’t want to get used to it (antihypertensive medication), and I decided I would stick with more natural methods, and sometimes I even felt better... After eating garlic sometimes I would go and check my blood pressure, and I found that my pressure was a bit lower.” (Female, 55 years)

Lifestyle modification that respondents had attempted included reducing salt, fats, oils, eating more fruits and vegetables and exercising. Among the 13 participants, only 2 stated they had definitive plans to follow up with a medical provider for their hypertension care.

**Lack of integration for hypertension and HIV care**

Participants reported that HIV and hypertension care were managed separately. Participants revealed that their HIV providers did not know they were hypertensive and that their hypertension providers also did not know they had HIV. Respondents did not discuss their HIV with their hypertension provider, did not discuss their hypertension with their HIV provider, and reported that their providers did not ask them if they had other medical issues or were taking other medications. The majority noted that their blood pressure was never measured in the HIV clinic. However, the HIV clinic was often the entry point into hypertension care. Respondents presented with symptoms to the HIV clinic, often during their regularly scheduled appointment, and were subsequently referred to the OPD for evaluation of their blood pressure. Two interviewees explained:

“When I went to pick up my medication (ART) they asked me how I was feeling. So I told them that I have these terrible headaches and I also feel dizzy. So they give me this piece of paper and they told me to go to the doctor and check my blood pressure at the OPD.” (Female, 42 years)

“No, I’ve never checked my blood pressure at the HIV clinic. There is not even a doctor at the HIV clinic. I’ve just seen nurses providing the HIV medication…If you tell them that you’re sick they tell you to go down there (pointing to OPD).” (Female, 39 years)

Although hypertension and HIV care were separate, all respondents conveyed a preference for integrated care due to convenience and efficiency.

“I think it will save a lot of time because I would get my (HIV) medication and then see the doctor right there (for hypertension). It would be very nice and I think would help improve my health and help me to feel a lot better without these headaches and dizziness.” (Female, 55 years)

“I would be so happy (to receive HIV and hypertension care in one location) because you may come today for one thing and a few days later another problem comes up. So, if they could check me for both things (at the same time) that would be really great.” (Female, 63 years)

**Additional barriers to hypertension care**

Additional barriers to hypertension care were described by participants. Financial barriers were the most common; participants described the high costs of medication, health insurance, provider visits, transport to the healthcare facility, and even the expense of a healthy lifestyle as making it difficult for them to manage their hypertension. A 78-year old woman described that the cost of medication was the reason for non-adherence.

“I'm not using (blood pressure medications)…it’s hard to raise the money now. You need money for food. You need money for this medication. If you suddenly get a fever you need money again, so how are you going to manage it?” (Female, 78 years)

Side effects due to antihypertensive drugs were also cited as a common reason for medication discontinuation. Participants who had never used blood pressure medication also expressed concern for potential side effects, toxicity to the body, and a fear of dependence on the medication as reasons why they would not want to initiate antihypertensive therapy.

“I’ve heard people say when you keep taking this (blood pressure) medication your body gets used to it, and it’s not a very good thing.” (Female, 63 years)

“When I take them (antihypertensive medications) I feel sleepy, so how can I take them every day? Who's going to take care of me? That's why I take them, and when I feel better, I stop.” (Female, 78 years)

System-level barriers were also noted. Participants described lack of access to medication as a reason for non-adherence. All participants who reported receiving a prescription for antihypertensive medication stated that they were given the prescription for only 5 to 7 days at a time, and also frequently reported pharmacy outages of the medication. A 55-year old woman stated:

“One of the biggest challenges with this hospital is that you may go and see the doctor, he may prescribe the medication, and you may be in this long queue, but when you get to the pharmacy they don’t have the medication. So, then you have to go to a private pharmacy outside (of the hospital) and they are a (private) business so you may have to pay 15,000 or 20,000 Tanzanian shillings (approximately 6 to 9 US dollars).” (Female, 55 years)

Some participants expressed frustration with the long wait times at health facilities which may have resulted from staff shortages. One participant stated:

“Maybe the only thing (challenge) is the delays in the clinic, because I have a lot of work that I need to do and sometimes there is a lot of people and so the line is quite long.” (Male, 73 years)

HIV stigma was an over-arching experience of participants. Many respondents reported feeling stigmatized from their HIV by their family and community and described a feeling of social isolation. A 44-year old woman described that she sometimes leaves her home due to stigmatization from her family.

“My brothers, at home, they stigmatize me a lot. Sometimes when I want to go to bed at night, they insult me the whole night. Or sometimes they take stones and throw them on the roof and I feel like I am shocked and fall down. So sometimes I don’t even stay at my home.” (Female, 44 years)

Stigmatization may have played a role in lower health seeking behaviors and led to HIV and hypertension nondisclosure to medical providers and subsequent disengagement in hypertension care.

**Discussion**

This study explored hypertension care perspectives and experiences among hypertensive adults engaged in HIV care in northern Tanzania. To our knowledge, only two other qualitative studies have previously described hypertension care experiences among patients with HIV in SSA [19,22]. In addition to challenges faced by the general population, respondents additionally mentioned issues specific to PLHIV including HIV-related stigma, and lack of integration between hypertension and HIV care. Of note, while participants in our study were disengaged in hypertension care, they remained completely engaged in HIV care. These findings suggest that the barriers depicted are unique to hypertension care and that the HIV clinic may provide a robust model of chronic disease management that can support retention in care. High quality, culturally salient, evidence-based interventions that are integrated within the HIV clinic are urgently needed to address the double burden of disease in PLHIV.

Poor and inaccurate hypertension knowledge among patients was identified as the predominant barrier to hypertension awareness, treatment and control in our study. Universally, all participants believed that ‘thinking too much’ was the major, if not sole, cause of their hypertension, and that one could control hypertension by ‘reducing thinking’. The concept of ‘thinking too much’ is an idiom of anxiety, depression and distress in many settings in SSA [30]. While anxiety may lead to a relative increase in blood pressure, it is not a major contributor of uncontrolled hypertension and stress reduction is not recognized as effective primary hypertension management [31,32]. Despite this, there was an ingrained belief in our sample that psychosocial challenges were a major contributor to hypertension risk and by reducing these psychosocial stressors one may control blood pressure. These findings, along with other limitations seen in the understanding of hypertension symptoms, complications, and treatment, are consistent with data on HIV-uninfected hypertensive patients from other studies in SSA [33–35]. In contrast, participants had good knowledge about HIV disease and treatment. The high levels of HIV care engagement seen among our participants may be due to concentrated efforts at patient-directed HIV education and highlights the importance of health education initiatives. Several studies in SSA have shown an improvement in hypertension outcomes following educational interventions. A patient education tool in Côte d'Ivoire reduced antihypertensive non-adherence from 60% to 5% one year after implementation [36], and in Ghana, receiving health education was found to be protective against uncontrolled hypertension [37]. Similar efforts to improve hypertension education should be implemented within the HIV clinical setting in order to improve cardiovascular outcomes among PLHIV. In addition, participants in our study reported mainly learning about hypertension from other members of the community, including hypertensive family members and friends. Given the success of peer education in HIV care in SSA [38–40] the role of peer educator in NCD management, including hypertension, should be strongly considered and further investigated.

Our findings suggest that hypertension management was limited by inadequate provider counseling and communication. Participants reported minimal counseling from providers about hypertension, which may have led to low levels of knowledge about the disease. This limited awareness of the silent, chronic nature of hypertension and effective hypertension treatment likely contributed to misconceptions, including false attributions of hypertension cause, and subsequent disengagement and loss to follow up. Similar studies in SSA have found that lack of counseling and gaps in knowledge and practice among healthcare providers are impediments to hypertension outcomes in the general population [34,41]. Provider counseling regarding all aspects of hypertension was lacking in our study. While providers did prescribe antihypertensive agents, medication counseling was insufficient and, at times, erroneous. Duration of treatment, side effects, and potential drug-drug interactions with ART were often not discussed, potentially leading to reluctance towards pharmacologic therapy and poor adherence to antihypertensive medications. Despite this, participants described their HIV medical providers as a source of education and support. This underscores the importance of patient-provider communication and the potential impact of improved provider counseling on health outcomes [42,43]. Health system interventions in HIV that have targeted providers have made considerable strides in improving HIV clinical outcomes in SSA. Similar interventions in hypertension training are essential to address the rising burden of CVD among PLHIV.

Unaffordable healthcare costs were identified by respondents as a driving factor towards hypertension disengagement and antihypertensive nonadherence. A study examining affordability and accessibility of antihypertensive drugs globally found that in SSA drugs were neither affordable nor accessible and that the cost of one tablet was often more than a day’s wages [44]. Unlike hypertension care, all aspects of HIV care are free in Tanzania, which likely plays a major role in ART uptake and long-term HIV care engagement. Universal health coverage is needed to support access to NCD care, but coverage must be affordable. Many participants in our study cited that they wanted health insurance but were unable to afford the high annual premiums. While the government has made major strides in improving health coverage, most Tanzanians remain uninsured [45]. As PLHIV are living longer and are at higher risk for other chronic diseases, enhanced coverage of NCDs in SSA must be addressed [2,46].

The participants in our study universally described high levels of HIV care engagement and ART adherence, and appeared highly knowledgeable and empowered in their HIV care. In contrast, no participant was adherent with their hypertension treatment or hypertension follow up, and hypertension knowledge was suboptimal. The barriers to hypertension care faced by PLHIV appear to be specific to hypertension, since these same barriers were not encountered in their HIV care. Of note, the barriers to hypertension care faced by PLHIV were actually quite similar to the barriers faced by the general population, however, PLHIV must also navigate HIV-related stigma and multiple siloed care systems to treat both their HIV and hypertension. Our findings are consistent with other qualitative studies exploring hypertension care barriers among PLHIV in similar settings [19,22], and signify the critical role of the HIV clinic. Given that patients are routinely engaged in and committed to their HIV care, this setting represents an opportunity to improve access of chronic disease care, beyond HIV, among PLHIV in SSA. Our findings suggest that integrating a horizontal model of healthcare with existing vertical HIV healthcare systems may expand access to NCD care and enhance patient satisfaction and clinical outcomes [47].

HIV and hypertension were managed in discrete silos in our setting, and our participants described the lack of integration as an impediment to their care. The separation of HIV and hypertension care represents a missed opportunity to improve cardiovascular outcomes among a high-risk population. Primary care and NCD models in SSA are either non-existent or are fragmented and weak. HIV is the first large chronic care program in SSA and has received significant investments. HIV clinics serve as models for robust service delivery and may provide a good opportunity to integrate NCD management. In 2011 the UN declared that HIV programs from low and middle-income countries should be leveraged for effective integration of NCDs [48,49]. Furthermore, the Tanzanian government has recognized the increased prevalence of NCDs among PLHIV and the potential critical role of the HIV clinic. Recent national guidelines in Tanzania now support the integration of care for HIV and other diseases including hypertension and other NCDs [50], and an increasing number of studies in SSA suggest that the integration of HIV and NCD care is feasible, efficacious and cost effective [49,51,52]. However, in order to achieve successful integration of the routine assessment, prevention and management of NCDS, including hypertension, it will be critical to address the system-level challenges and ensure access to trained staff, equipment, medications and protocols for NCD care within the HIV clinical setting [20,53].

This study has some limitations. Firstly, inclusion criteria for hypertension was based on a self-reported diagnosis instead of a clinical diagnosis. Therefore, it is possible that some participants were not truly hypertensive, and thus fittingly did not engage in hypertension care. Secondly, social desirability bias when speaking with a non-Tanzanian physician may have influenced participants’ responses. Thirdly, our recruitment strategy excluded patients who had dropped out of HIV care. Therefore, we may have lost some additional insight into perspectives and experiences of an HIV-disengaged population. Fourthly, we also recognize that this is a relatively small qualitative sample size [54]. Although our analysis strategy identified data saturation by participant number 13, it is possible that recruitment from a larger sample may have yielded additional emerging themes. Lastly, only two men participated in this study and therefore findings may be biased towards a female perspective. The challenge of recruiting men is not unique to our study. Men may be less likely to seek healthcare and remain engaged in care in SSA [55,56]. Additional research to explore male perspectives of hypertension care is needed.

**Conclusions**

In conclusion, hypertensive adults engaged in HIV care in northern Tanzania described multiple, intersecting challenges to hypertension management. Multifaceted strategies that improve hypertension education and counseling, and that are affordable, accessible and integrated within HIV care are urgently needed to improve cardiovascular outcomes among PLHIV. As more individuals with HIV initiate ART and continue to age, the prevalence of hypertension and its associated cardiovascular morbidity and mortality among PLHIV will almost certainly continue to rise. Thus, it is imperative to develop and implement effective strategies for integrating cardiovascular care in the HIV clinical setting.

**Acknowledgements**

We would like to thank the Duke Global Health Institute, the Duke Hubert-Yeargan Center for Global Health, and all the staff of the Pasua Health Center, Majengo Health Center, Kilimanjaro Clinical Research Institute and the KCMC-Duke Collaboration for all their efforts and support for this study. We give special thanks to Pilli Nyindo for her integral role in recruiting respondents for this study.

**References**

[1]	Frank TD, Carter A, Jahagirdar D, Biehl MH, Douwes-Schultz D, Larson SL, et al. Global, regional, and national incidence, prevalence, and mortality of HIV, 1980–2017, and forecasts to 2030, for 195 countries and territories: a systematic analysis for the Global Burden of Diseases, Injuries, and Risk Factors Study 2017. The Lancet HIV 2019;0. https://doi.org/10.1016/S2352-3018(19)30196-1.

[2]	Patel P, Rose CE, Collins PY, Nuche-Berenguer B, Sahasrabuddhe VV, Peprah E, et al. Noncommunicable diseases among HIV-infected persons in low-income and middle-income countries: a systematic review and meta-analysis. AIDS 2018;32 Suppl 1:S5–20. https://doi.org/10.1097/QAD.0000000000001888.

[3]	Feinstein MJ, Hsue PY, Benjamin LA, Bloomfield GS, Currier JS, Freiberg MS, et al. Characteristics, Prevention, and Management of Cardiovascular Disease in People Living With HIV: A Scientific Statement From the American Heart Association. Circulation 2019;140:e98–124. https://doi.org/10.1161/CIR.0000000000000695.

[4]	Roth GA, Abate D, Abate KH, Abay SM, Abbafati C, Abbasi N, et al. Global, regional, and national age-sex-specific mortality for 282 causes of death in 195 countries and territories, 1980–2017: a systematic analysis for the Global Burden of Disease Study 2017. The Lancet 2018;392:1736–88. https://doi.org/10.1016/S0140-6736(18)32203-7.

[5]	Tseng ZH, Secemsky EA, Dowdy D, Vittinghoff E, Moyers B, Wong JK, et al. Sudden cardiac death in patients with human immunodeficiency virus infection. J Am Coll Cardiol 2012;59:1891–6. https://doi.org/10.1016/j.jacc.2012.02.024.

[6]	Shah ASV, Stelzle D, Lee KK, Beck EJ, Alam S, Clifford S, et al. Global Burden of Atherosclerotic Cardiovascular Disease in People Living With HIV. Circulation 2018;138:1100–12. https://doi.org/10.1161/CIRCULATIONAHA.117.033369.

[7]	GBD 2016 Risk Factors Collaborators. Global, regional, and national comparative risk assessment of 84 behavioural, environmental and occupational, and metabolic risks or clusters of risks, 1990-2016: a systematic analysis for the Global Burden of Disease Study 2016. Lancet 2017;390:1345–422. https://doi.org/10.1016/S0140-6736(17)32366-8.

[8]	Xu Y, Chen X, Wang K. Global prevalence of hypertension among people living with HIV: a systematic review and meta-analysis. J Am Soc Hypertens 2017;11:530–40. https://doi.org/10.1016/j.jash.2017.06.004.

[9]	WHO | Raised blood pressure. WHO n.d. https://www.who.int/gho/ncd/risk_factors/blood_pressure_prevalence_text/en/ (accessed September 24, 2019).

[10]	Geldsetzer P, Manne-Goehler J, Marcus M-E, Ebert C, Zhumadilov Z, Wesseh CS, et al. The state of hypertension care in 44 low-income and middle-income countries: a cross-sectional study of nationally representative individual-level data from 1·1 million adults. The Lancet 2019;394:652–62. https://doi.org/10.1016/S0140-6736(19)30955-9.

[11]	Ataklte F, Erqou S, Kaptoge S, Taye B, Echouffo-Tcheugui JB, Kengne AP. Burden of undiagnosed hypertension in sub-saharan Africa: a systematic review and meta-analysis. Hypertension 2015;65:291–8. https://doi.org/10.1161/HYPERTENSIONAHA.114.04394.

[12]	Peck RN, Shedafa R, Kalluvya S, Downs JA, Todd J, Suthanthiran M, et al. Hypertension, kidney disease, HIV and antiretroviral therapy among Tanzanian adults: a cross-sectional study. BMC Med 2014;12:125. https://doi.org/10.1186/s12916-014-0125-2.

[13]	Manavalan P, Madut DB, Hertz JT, Thielman NM, Okeke NL, Mmbaga BT, et al. Hypertension burden and challenges across the hypertension treatment cascade among adults enrolled in HIV care in northern Tanzania. J Clin Hypertens 2020:Forthcoming. https://doi.org/10.1111/jch.13929.

[14]	Peck R, Mghamba J, Vanobberghen F, Kavishe B, Rugarabamu V, Smeeth L, et al. Preparedness of Tanzanian health facilities for outpatient primary care of hypertension and diabetes: a cross-sectional survey. Lancet Glob Health 2014;2:e285-292. https://doi.org/10.1016/S2214-109X(14)70033-6.

[15]	Musinguzi G, Bastiaens H, Wanyenze RK, Mukose A, Van Geertruyden J-P, Nuwaha F. Capacity of Health Facilities to Manage Hypertension in Mukono and Buikwe Districts in Uganda: Challenges and Recommendations. PLoS ONE 2015;10:e0142312. https://doi.org/10.1371/journal.pone.0142312.

[16]	Chang H, Hawley NL, Kalyesubula R, Siddharthan T, Checkley W, Knauf F, et al. Challenges to hypertension and diabetes management in rural Uganda: a qualitative study with patients, village health team members, and health care professionals. Int J Equity Health 2019;18:38. https://doi.org/10.1186/s12939-019-0934-1.

[17]	Kane JC, Elafros MA, Murray SM, Mitchell EMH, Augustinavicius JL, Causevic S, et al. A scoping review of health-related stigma outcomes for high-burden diseases in low- and middle-income countries. BMC Med 2019;17:17. https://doi.org/10.1186/s12916-019-1250-8.

[18]	Rice WS, Crockett KB, Mugavero MJ, Raper JL, Atkins GC, Turan B. Association Between Internalized HIV-Related Stigma and HIV Care Visit Adherence. J Acquir Immune Defic Syndr 2017;76:482–7. https://doi.org/10.1097/QAI.0000000000001543.

[19]	Temu TM, Bahiru E, Bukachi F, Bloomfield GS, Muiruri P, Farquhar C. Lay beliefs about hypertension among HIV-infected adults in Kenya. Open Heart 2017;4:e000570. https://doi.org/10.1136/openhrt-2016-000570.

[20]	Leung C, Aris E, Mhalu A, Siril H, Christian B, Koda H, et al. Preparedness of HIV care and treatment clinics for the management of concomitant non-communicable diseases: a cross-sectional survey. BMC Public Health 2016;16:1002. https://doi.org/10.1186/s12889-016-3661-1.

[21]	Kagaruki GB, Mayige MT, Ngadaya ES, Kilale AM, Kahwa A, Shao AF, et al. Knowledge and perception on type2 diabetes and hypertension among HIV clients utilizing care and treatment services: a cross sectional study from Mbeya and Dar es Salaam regions in Tanzania. BMC Public Health 2018;18. https://doi.org/10.1186/s12889-018-5639-7.

[22]	Hing M, Hoffman RM, Seleman J, Chibwana F, Kahn D, Moucheraud C. ‘Blood pressure can kill you tomorrow, but HIV gives you time’: illness perceptions and treatment experiences among Malawian individuals living with HIV and hypertension. Health Policy Plan 2019;34:ii36–44. https://doi.org/10.1093/heapol/czz112.

[23]	Weiss JJ, Konstantinidis I, Boueilh A, Fierer DS, Gardenier D, Barber MG, et al. Illness Perceptions, Medication Beliefs, and Adherence to Antiretrovirals and Medications for Comorbidities in Adults with HIV Infection and Hypertension or Chronic Kidney Disease. J Acquir Immune Defic Syndr 2016;73:403–10. https://doi.org/10.1097/QAI.0000000000001075.

[24]	Tong A, Sainsbury P, Craig J. Consolidated criteria for reporting qualitative research (COREQ): a 32-item checklist for interviews and focus groups. Int J Qual Health Care 2007;19:349–57. https://doi.org/10.1093/intqhc/mzm042.

[25]	Galson SW, Staton CA, Karia F, Kilonzo K, Lunyera J, Patel UD, et al. Epidemiology of hypertension in Northern Tanzania: a community-based mixed-methods study. BMJ Open 2017;7:e018829. https://doi.org/10.1136/bmjopen-2017-018829.

[26]	Guest G, Macqueen K, Namey E. Applied Thematic Analysis. 2012. https://doi.org/10.4135/9781483384436.

[27]	Smith JA, Osborn M. Interpretative Phenomenological Analysis. Thousand Oaks: Sage Publications; 2008.

[28]	Davidsen AS. Phenomenological Approaches in Psychology and Health Sciences. Qual Res Psychol 2013;10:318–39. https://doi.org/10.1080/14780887.2011.608466.

[29]	Birks M, Chapman Y, Francis K. Memoing in qualitative research: Probing data and processes. Journal of Research in Nursing 2008;13:68–75. https://doi.org/10.1177/1744987107081254.

[30]	Kaiser BN, Haroz EE, Kohrt BA, Bolton PA, Bass JK, Hinton DE. “Thinking too much”: A Systematic review of a common idiom of distress. Soc Sci Med 2015;147:170–83. https://doi.org/10.1016/j.socscimed.2015.10.044.

[31]	Brook Robert D., Appel Lawrence J., Rubenfire Melvyn, Ogedegbe Gbenga, Bisognano John D., Elliott William J., et al. Beyond Medications and Diet: Alternative Approaches to Lowering Blood Pressure. Hypertension 2013;61:1360–83. https://doi.org/10.1161/HYP.0b013e318293645f.

[32]	Whelton PK, Carey RM, Aronow WS, Casey DE, Collins KJ, Himmelfarb CD, et al. 2017 ACC/AHA/AAPA/ABC/ACPM/AGS/APhA/ASH/ASPC/NMA/PCNA Guideline for the Prevention, Detection, Evaluation, and Management of High Blood Pressure in Adults: A Report of the American College of Cardiology/American Heart Association Task Force on Clinical Practice Guidelines. J Am Coll Cardiol 2018;71:e127–248. https://doi.org/10.1016/j.jacc.2017.11.006.

[33]	Nyaaba GN, Masana L, Aikins A de-Graft, Stronks K, Agyemang C. Lay community perceptions and treatment options for hypertension in rural northern Ghana: a qualitative analysis. BMJ Open 2018;8:e023451. https://doi.org/10.1136/bmjopen-2018-023451.

[34]	Murphy K, Chuma T, Mathews C, Steyn K, Levitt N. A qualitative study of the experiences of care and motivation for effective self-management among diabetic and hypertensive patients attending public sector primary health care services in South Africa. BMC Health Serv Res 2015;15:303. https://doi.org/10.1186/s12913-015-0969-y.

[35]	Taylor KD, Adedokun A, Awobusuyi O, Adeniran P, Onyia E, Ogedegbe G. Explanatory models of hypertension among Nigerian patients at a university teaching hospital. Ethn Health 2012;17:615–29. https://doi.org/10.1080/13557858.2013.771151.

[36]	Koffi J, Konin C, Gnaba A, NGoran Y, Mottoh N, Guikahue MK. [Usefulness of patient education in antihypertensive treatment compliance in black Africans]. Ann Cardiol Angeiol (Paris) 2018;67:9–13. https://doi.org/10.1016/j.ancard.2017.04.012.

[37]	Goverwa TP, Masuka N, Tshimanga M, Gombe NT, Takundwa L, Bangure D, et al. Uncontrolled hypertension among hypertensive patients on treatment in Lupane District, Zimbabwe, 2012. BMC Res Notes 2014;7:703. https://doi.org/10.1186/1756-0500-7-703.

[38]	Menna T, Ali A, Worku A. Effects of peer education intervention on HIV/AIDS related sexual behaviors of secondary school students in Addis Ababa, Ethiopia: a quasi-experimental study. Reprod Health 2015;12:84. https://doi.org/10.1186/s12978-015-0077-9.

[39]	Tapera T, Willis N, Madzeke K, Napei T, Mawodzeke M, Chamoko S, et al. Effects of a Peer-Led Intervention on HIV Care Continuum Outcomes Among Contacts of Children, Adolescents, and Young Adults Living With HIV in Zimbabwe. Glob Health Sci Pract 2019;7:575–84. https://doi.org/10.9745/GHSP-D-19-00210.

[40]	Laukamm-Josten U, Mwizarubi BK, Outwater A, Mwaijonga CL, Valadez JJ, Nyamwaya D, et al. Preventing HIV infection through peer education and condom promotion among truck drivers and their sexual partners in Tanzania, 1990-1993. AIDS Care 2000;12:27–40. https://doi.org/10.1080/09540120047440.

[41]	Lulebo AM, Mapatano MA, Kayembe PK, Mafuta EM, Mutombo PB, Coppieters Y. Assessment of hypertension management in primary health care settings in Kinshasa, Democratic Republic of Congo. BMC Health Serv Res 2015;15:573. https://doi.org/10.1186/s12913-015-1236-y.

[42]	Gyamfi J, Plange-Rhule J, Iwelunmor J, Lee D, Blackstone SR, Mitchell A, et al. Training nurses in task-shifting strategies for the management and control of hypertension in Ghana: a mixed-methods study. BMC Health Serv Res 2017;17:104. https://doi.org/10.1186/s12913-017-2026-5.

[43]	Ogola EN, Okello FO, Herr JL, Macgregor-Skinner E, Mulvaney A, Yonga G. Healthy Heart Africa-Kenya: A 12-Month Prospective Evaluation of Program Impact on Health Care Providers’ Knowledge and Treatment of Hypertension. Glob Heart 2019;14:61–70. https://doi.org/10.1016/j.gheart.2019.02.002.

[44]	van Mourik MS, Cameron A, Ewen M, Laing RO. Availability, price and affordability of cardiovascular medicines: A comparison across 36 countries using WHO/HAI data. BMC Cardiovasc Disord 2010;10:25. https://doi.org/10.1186/1471-2261-10-25.

[45]	United Republic of Tanzania | Universal Health Coverage Partnership n.d. https://uhcpartnership.net/country-profile/tanzania/ (accessed September 22, 2019).

[46]	Gouda HN, Charlson F, Sorsdahl K, Ahmadzada S, Ferrari AJ, Erskine H, et al. Burden of non-communicable diseases in sub-Saharan Africa, 1990–2017: results from the Global Burden of Disease Study 2017. The Lancet Global Health 2019;7:e1375–87. https://doi.org/10.1016/S2214-109X(19)30374-2.

[47]	Mercer T, Chang AC, Fischer L, Gardner A, Kerubo I, Tran DN, et al. Mitigating The Burden Of Diabetes In Sub-Saharan Africa Through An Integrated Diagonal Health Systems Approach. Diabetes Metab Syndr Obes 2019;12:2261–72. https://doi.org/10.2147/DMSO.S207427.

[48]	UNAIDS | Chronic Care of HIV and Noncommunicable Diseases n.d. https://www.unaids.org/sites/default/files/media_asset/20110526_JC2145_Chronic_care_of_HIV_0.pdf (accessed September 22, 2019).

[49]	Patel P, Speight C, Maida A, Loustalot F, Giles D, Phiri S, et al. Integrating HIV and hypertension management in low-resource settings: Lessons from Malawi. PLoS Med 2018;15. https://doi.org/10.1371/journal.pmed.1002523.

[50]	Tanzanian Ministry of Health, Community Development, Gender, Elderly and Children | HIV Service Delivery Models: Mapping HIV service delivery strategies in Tanzania n.d. [http://www.differentiatedcare.org/Portals/0/adam/Content/nKMF99s69UqVMa9DJQp96A/File/SDM_Tanzania_Mapping Report_04pril2017_FINAL.pdf](http://www.differentiatedcare.org/Portals/0/adam/Content/nKMF99s69UqVMa9DJQp96A/File/SDM_Tanzania_Mapping%20Report_04pril2017_FINAL.pdf). (accessed June 29, 2020).

[51]	Kwarisiima D, Atukunda M, Owaraganise A, Chamie G, Clark T, Kabami J, et al. Hypertension control in integrated HIV and chronic disease clinics in Uganda in the SEARCH study. BMC Public Health 2019;19:511. https://doi.org/10.1186/s12889-019-6838-6.

[52]	Sando D, Kintu A, Okello S, Kawungezi PC, Guwatudde D, Mutungi G, et al. Cost-effectiveness analysis of integrating screening and treatment of selected non-communicable diseases into HIV/AIDS treatment in Uganda. J Int AIDS Soc 2020;23 Suppl 1:e25507. https://doi.org/10.1002/jia2.25507.

[53]	Pfaff C, Scott V, Hoffman R, Mwagomba B. You can treat my HIV - But can you treat my blood pressure? Availability of integrated HIV and non-communicable disease care in northern Malawi. Afr J Prim Health Care Fam Med 2017;9:e1–8. https://doi.org/10.4102/phcfm.v9i1.1151.

[54]	Guest G, Bunce A, Johnson L. How Many Interviews Are Enough?: An Experiment with Data Saturation and Variability. Field Methods 2016. https://doi.org/10.1177/1525822X05279903.

[55]	Camlin CS, Ssemmondo E, Chamie G, El Ayadi AM, Kwarisiima D, Sang N, et al. Men “missing” from population-based HIV testing: insights from qualitative research. AIDS Care 2016;28 Suppl 3:67–73. https://doi.org/10.1080/09540121.2016.1164806.

[56]	Sharma M, Barnabas RV, Celum C. Community-based strategies to strengthen men’s engagement in the HIV care cascade in sub-Saharan Africa. PLoS Med 2017;14. https://doi.org/10.1371/journal.pmed.1002262.

**Supporting information**

**S1 File. COREQ Checklist.**

**S2 File. In-depth Interview Guide.**
