# Supplementary material for: “It’s because I think too much”: Perspectives and experiences of adults with hypertension engaged in HIV care in northern Tanzania
Source: PLoS One. 2020 Dec 3;15(12):e0243059. doi: 10.1371/journal.pone.0243059 (PMC7714125; doi:10.1371/journal.pone.0243059)
Supplement: Attachment — Submitted filename: Response to Reviewers.docx [file pone.0243059.s005.docx]

September 24, 2020

The Editor

*PLOS ONE*

**Re: PONE-D-20-04882, “It’s because I think too much”: Perspectives, experiences and opportunities for improvement among adults with hypertension engaged in HIV care in northern Tanzania**

Dear Dr. Webster Mavhu,

Thank you again for the opportunity to provide a revision for this manuscript. We have addressed each comment and suggestion. The specific nature of the response to each point is outlined with our submission and changes are made, where appropriate, in the revised manuscript text. In particular, we have focused on issues specific to people living with HIV by revising the results and discussion to highlight the key barriers to hypertension care faced by people living with HIV. To our knowledge, there have been few qualitative manuscripts published in the literature exploring hypertension care experiences among people living with HIV in sub-Saharan Africa, and we believe our findings contribute to the literature supporting integrated HIV and chronic disease health services.

This manuscript has not been previously published nor is it under consideration for publication elsewhere. The authors have seen and approved the version of the manuscript enclosed and concur with its submission to *PLOS ONE*.

Thank you very much for considering this revision.

Sincerely,

Preeti Manavalan, MD, MSc-GH

Assistant Professor of Medicine

Division of Infectious Diseases and Global Medicine

University of Florida

**Academic Editor**

1. **Comment**: The paper has significantly improved.

**Response**: We thank the editor for this positive feedback and have addressed specific comments and critical feedback below.

2. **Comment**: The paper discusses a lot of general issues but could focus only on those related to PLHIV - paper could come down to 3,500 words.

**Response**: We thank the editor for this thoughtful feedback. We have carefully reviewed the manuscript with a view to tightening the text and focusing more on the issues unique to people living with HIV. We were able to reduce the text substantially, shortening from 6668 words to 4688 words. We felt that reducing the word count further may remove pertinent content and would delete content that reviewers had asked for in the initial review. The revised results now focus primarily on barriers experienced uniquely by people living with HIV. In addition, revisions to the discussion have been made to similarly focus on themes unique to people living with HIV (e.g., HIV-related stigma, burden from having multiple medical conditions, and siloed health care). We have also added the following paragraph to the discussion to highlight the significance of HIV-related stigma, stress and mental health; in doing so, we hope to make the added value of this study more apparent and bring more nuance to the paper.

“Stress and HIV-related stigma were overarching themes described by all participants in our study. Participants believed that stress related to living with HIV caused hypertension. They also described feeling overwhelmed and “burdened” from having more than one medical condition. Moreover, some participants described experiences of HIV-related stigma which may have led to additional stress, anxiety, depression and social isolation. Chronic untreated mental health conditions and HIV-related stigma are well-known predictors of ART nonadherence and poor HIV clinical outcomes [39–41]. In addition, stigma avoidant behaviors, i.e. those actions patients take to avoid experiencing stigma such as concealing ART or non-disclosure of HIV status, may also contribute to nonadherence and disengagement with the medical system [42]. Furthermore, evidence suggests that chronic mental health conditions such as depression may also be a predictor for hypertension treatment nonadherence in similar settings [43]. Therefore, it is possible that psychosocial stressors, disease-related stigma and stigma-avoidant behaviors contributed to hypertension care disengagement and nonadherence in our sample. Further exploration of mental health conditions, stigma and their effects on clinical outcomes in HIV and other chronic diseases among PLHIV in SSA should be prioritized to better understand the factors contributing to care disengagement. In addition, due to stigmatization, patients with HIV may feel more comfortable navigating NCD care within the HIV clinical setting with providers in which an established and trusting relationship already exists. The effects of integrating health care on HIV-related stigma and mental health outcomes among PLHIV should also be further investigated.” (page 19, paragraph 2)

As we have substantially shortened this manuscript to focus on issues specific to people living with HIV, we have removed from the discussion reflections upon barriers that might be common across the general population of individuals with hypertension.

3. **Comment**: Could also reduce quotes - maximum of two per point (and select the powerful ones).

**Response**: We thank the editor for this feedback. We have reduced the number of quotes in the results section and have selected quotes that focus on barriers unique to people living with HIV.

4. **Comment**: Results could be re-arranged.

**Response**: We thank the editor for this feedback. We have re-arranged the results so that the previous theme ‘Limited understanding of the causes of hypertension’ has been removed and is now discussed within the theme ‘Limited knowledge of hypertension’. In addition, we have revised the theme ‘Multiple barriers to hypertension care’ to ‘Additional structural barriers to hypertension care’ and have revised the subthemes within this theme to better reflect this change. The subthemes of ‘Additional structural barriers to hypertension care’ now include the following: High cost of care, Lack of access to antihypertensive medication, Staff shortages and long wait times, and HIV-associated stigma and social isolation.

5. **Comment**: I am still struggling with some sections which mention lack of knowledge, but later show participants had knowledge. Perhaps a more nuanced way of looking at this would be to say, 'When asked about what they were doing to reduce risk of hypertension, only 1 participant mentioned that they were taking less salt, fats, oil. However, when asked about the knowledge they had received from providers around this aspect, they mentioned several mitigation strategies, suggesting a mismatch between knowledge and practice' or something similar.

**Response**: We thank the editor for this comment. We have revised the results to reflect that knowledge about some aspects of hypertension were mixed and have added the following statement: “Knowledge regarding unhealthy lifestyle behaviors was mixed. All but one participant identified a diet high in salt and fat as a cause for hypertension. Few participants identified other unhealthy behaviors, including sedentary lifestyle, obesity, and alcohol and tobacco use as a cause.” (page 11, lines 181-184). In addition, as we have substantially shortened the manuscript to focus more on barriers specific to people living with HIV and less on barriers among the general population, we have removed from the results knowledge received from providers around lifestyle modification counseling.

6. **Comment**: See additional suggestions in manuscript itself.

**Response**: We thank the editor for the additional edits and suggestions in the manuscript itself. We have accepted the editor’s edits. We have included the comments in the track-changes version of the manuscript and included our responses as comments.
