# Supplementary material for: “It’s because I think too much”: Perspectives and experiences of adults with hypertension engaged in HIV care in northern Tanzania
Source: PLoS One. 2020 Dec 3;15(12):e0243059. doi: 10.1371/journal.pone.0243059 (PMC7714125; doi:10.1371/journal.pone.0243059)
Supplement: Attachment — Submitted filename: Response to Reviewers.docx [file pone.0243059.s007.docx]

November 12, 2020

The Editor

*PLOS ONE*

**Re: PONE-D-20-04882R2, “It’s because I think too much”: Perspectives and experiences of adults with hypertension engaged in HIV care in northern Tanzania**

Dear Dr. Webster Mavhu,

Thank you again for the opportunity to provide a revision for this manuscript. We have addressed the few suggested edits and all changes have been made in the revised manuscript text. Please find below a detailed response to all comments and concerns. To our knowledge, there have been few qualitative manuscripts published in the literature exploring hypertension care experiences among people living with HIV in sub-Saharan Africa. We believe our findings contribute to the literature supporting integrated HIV and chronic disease health services, and may help address barriers to cardiovascular disease care among people living with HIV.

Thank you very much for considering this revision.

Sincerely,

Preeti Manavalan, MD, MSc-GH

Assistant Professor of Medicine

Division of Infectious Diseases and Global Medicine

University of Florida

**Academic Editor**

1. **Comment**: We will accept the manuscript once the few suggested edits are addressed.

**Response**: We thank the editor for this positive feedback and for the additional edits in the manuscript itself. We have accepted all of the editor’s suggestions and edits.
